# *Relictithismia kimotsukiensis,* a new genus and species of Thismiaceae from southern Japan with discussions on its phylogenetic relationship

**DOI:** 10.1007/s10265-024-01532-5

**Published:** 2024-02-29

**Authors:** Kenji Suetsugu, Yasunori Nakamura, Takafumi Nakano, Shuichiro Tagane

**Affiliations:** 1 Department of Biology, Graduate School of Science, Kobe University, Kobe, Hyogo 657-8501 Japan; 2 Institute for Advanced Research, Kobe University, 1-1 Rokkodai, Nada-Ku, Kobe, Hyogo 657-8501 Japan; 3 Tajiri, Nishi-Ku, Fukuoka, Fukuoka 819-0383 Japan; 4 Department of Zoology, Graduate School of Science, Kyoto University, Sakyo-Ku, Kyoto, 606-8502 Japan; 5 Kagoshima University Museum, Kagoshima University, 1-21-30, Korimoto, Kagoshima, 890-0065 Japan

**Keywords:** Angiosperms, Endemic, Flora, Dioscoreales, East Asia, Taxonomy

## Abstract

The family Thismiaceae, known as “fairy lanterns” for their urn- or bell-shaped flowers with basally fused tepals, consists of non-photosynthetic flowering monocots mainly in tropical regions, extending into subtropical and temperate areas. Here, we propose a new mycoheterotrophic genus, *Relictithismia* Suetsugu & Tagane (Thismiaceae), with its monotypic species *Relictithismia kimotsukiensis* Suetsugu, Yas.Nakam. & Tagane from Kimotsuki Mountains in the Osumi Peninsula, Kagoshima Prefecture, Kyushu Island, southern Japan. *Relictithismia* resembles *Haplothismia* Airy Shaw in having a cluster of tuberous roots, a feature previously observed only in this genus within the family Thismiaceae. However, it differs in having solitary flowers (vs. 2–6-flowered pseudo-raceme in *Haplothismia*), anther thecae largely separated (vs. connate), and the presence of an annulus (vs. absent). Additionally, *Relictithismia* differs from the geographically overlapping genus *Thismia* Griff. in its stamen structure and the position of the annulus. In *Relictithismia*, the stamens lack connectives, and its free filaments arise from the annulus located inside the perianth mouth, while in *Thismia*, the stamens typically have connate connectives, forming a staminal tube pendulous from the annulus located at the mouth of the floral tube. Our morphological and phylogenetic data indicated that *R. kimotsukiensis* holds an early-diverging position within the family, situated outside the Old World *Thismia* clade. This paper offers an extensive description and color photographs of *R. kimotsukiensis*, complemented by notes on its phylogenetic relationship and evolutionary history.

**Supplementary Information:**

The online version contains supplementary material available at 10.1007/s10265-024-01532-5.

## Introduction

The family Thismiaceae J.Agardh, commonly referred to as “fairy lanterns” for their vibrant, urn- or bell-shaped flowers with basally fused tepals, consists of non-photosynthetic flowering monocots primarily found in tropical regions, with extensions into subtropical and temperate areas (Garrett et al. 2023; Merckx and Smets 2014; Thorogood and Mat Yunoh 2021). Most studies considered that this family encompasses the monotypic genera *Haplothismia* Airy Shaw and *Tiputinia* P.E.Berry & C.L.Woodw., *Oxygyne* Schltr. with six species, *Thismia* Griff. with about 100 species, and *Afrothismia* Schltr., thought to have around 16 species (Merckx et al. 2006, 2009; POWO 2024; Shepeleva et al. 2020), although recent phylogenetic research indicates *Afrothismia* probably does not belong to the family Thismiaceae and is proposed to be an independent family (Cheek et al. 2023).

Mycoheterotrophic plants, which typically obtain their carbon indirectly from photosynthetic plants through shared mycorrhizal networks, depend heavily on the symbiotic interactions between fungi and trees (Gomes et al. 2017; Suetsugu et al. 2022b). This complex dependency renders them particularly susceptible to environmental disturbances, often leading to their rarity and endangered status. Members of the family Thismiaceae are especially notable for their exceptional scarcity and highly localized habitats (Suetsugu et al. 2023; Thorogood 2019; Thorogood and Mat Yunoh 2021). For instance, out of the approximately 100 described *Thismia* species, at least 55 are known only from their original discovery locations, and at least 38 have been documented based on a single discovery instance (Dančák et al. 2020).

The taxonomy of mycoheterotrophic plants has been a subject of considerable debate, largely due to the significant reduction in vegetative organs, their rarity, and the high rates of molecular evolution. The classification of Thismiaceae, in particular, has been contested. The APG group placed Thismiaceae within Burmanniaceae Blume (The Angiosperm Phylogeny Group 2016), a decision that may have been influenced by contaminant sequences (Lam 2016). Other studies using Sanger sequencing of specific mitochondrial and nuclear genes have underscored the distinctiveness of Thismiaceae from Burmanniaceae (Merckx et al. 2006, 2009; Shepeleva et al. 2020). These comprehensive analyses have positioned Thismiaceae *s.s.* (specifically, *Thismia*, *Tiputinia*, *Haplothismia*, and *Oxygyne*) as a sister group to Taccaceae Dumort., placing them in a separate subclade from Burmanniaceae within the order Dioscoreales. The recognition of Thismiaceae as a distinct family is further supported by recent phylogenomic studies that focus on high-throughput plastid and mitochondrial data (Garrett et al. 2023; Lam et al. 2018; Lin et al. 2022), and are complemented by morphological data (Cheek et al. 2018; Merckx et al. 2006).

Moreover, several phylogenetic studies indicated that *Afrothismia*, typically classified under Thismiaceae, is not monophyletic with the other four genera but is instead a sister clade with photosynthetic Taccaceae and other Thismiaceae species (Lin et al. 2022; Merckx et al. 2009). Morphologically, *Afrothismia* is uniquely characterized by its underground system comprising short rhizome bearing clusters of globose bulbils, each with a terminal rootlet, distinct from the coralloid, vermiform, or tuberous roots typical of other Thismiaceae species (Cheek et al. 2023; Merckx et al. 2009). Furthermore, the ovary in *Afrothismia* is characterized by having a single stalked placenta (Cheek et al. 2023). This feature distinguishes it from other species in the family Thismiaceae, which have three placentae. These insights have led to proposals for reclassifying *Afrothismia* as a distinct family (Cheek et al. 2023). An alternate approach of grouping *Afrothismia* (alongside Taccaceae and Thismiaceae) within a broadly defined Dioscoreaceae R.Br. may mask the significant morphological diversity among these families. Classifying a wide range of morphologies under Dioscoreaceae could make it undiagnosable due to high internal variation (Cheek et al. 2023). Thus, recognizing *Afrothismia* at the family level seems to be justifiable. However, due to their apparent morphological similarities, *Afrothismia* has been included in a morphological comparison with our newly described genus in this paper.

The Japanese archipelago, extending from boreal to subtropical zones, is a crucial part of the continental islands in East Asia (Kubota et al. 2017). It has been proposed as a refuge for Tertiary relict floras, harboring ancient plant species (Milne and Abbott 2002). The present biotas of the archipelago are shaped by a history of migrations, extinctions, and speciations, influenced by geographical isolation and connections (Millien-Parra and Jaeger 1999). This unique configuration makes the Japanese archipelago an ideal region for studying the impact of geohistorical processes on floristic diversification (Kubota et al. 2017). Furthermore, Japanese flora is notably rich in arbuscular mycorrhizal (AM) mycoheterotrophic plants (Suetsugu and Nishioka 2017; Suetsugu et al. 2023). To date, over 20 AM mycoheterotrophic species from four different families (Petrosaviaceae Hutch., Triuridaceae Gardner, Burmanniaceae, and Thismiaceae) have been documented. The Japanese species often represent the northernmost members of their genera or families (Suetsugu et al. 2023). Thus, the Japanese AM mycoheterotrophic plants provide essential insights into the biogeography and evolutionary history of these elusive plants.

**Fig. 1:**
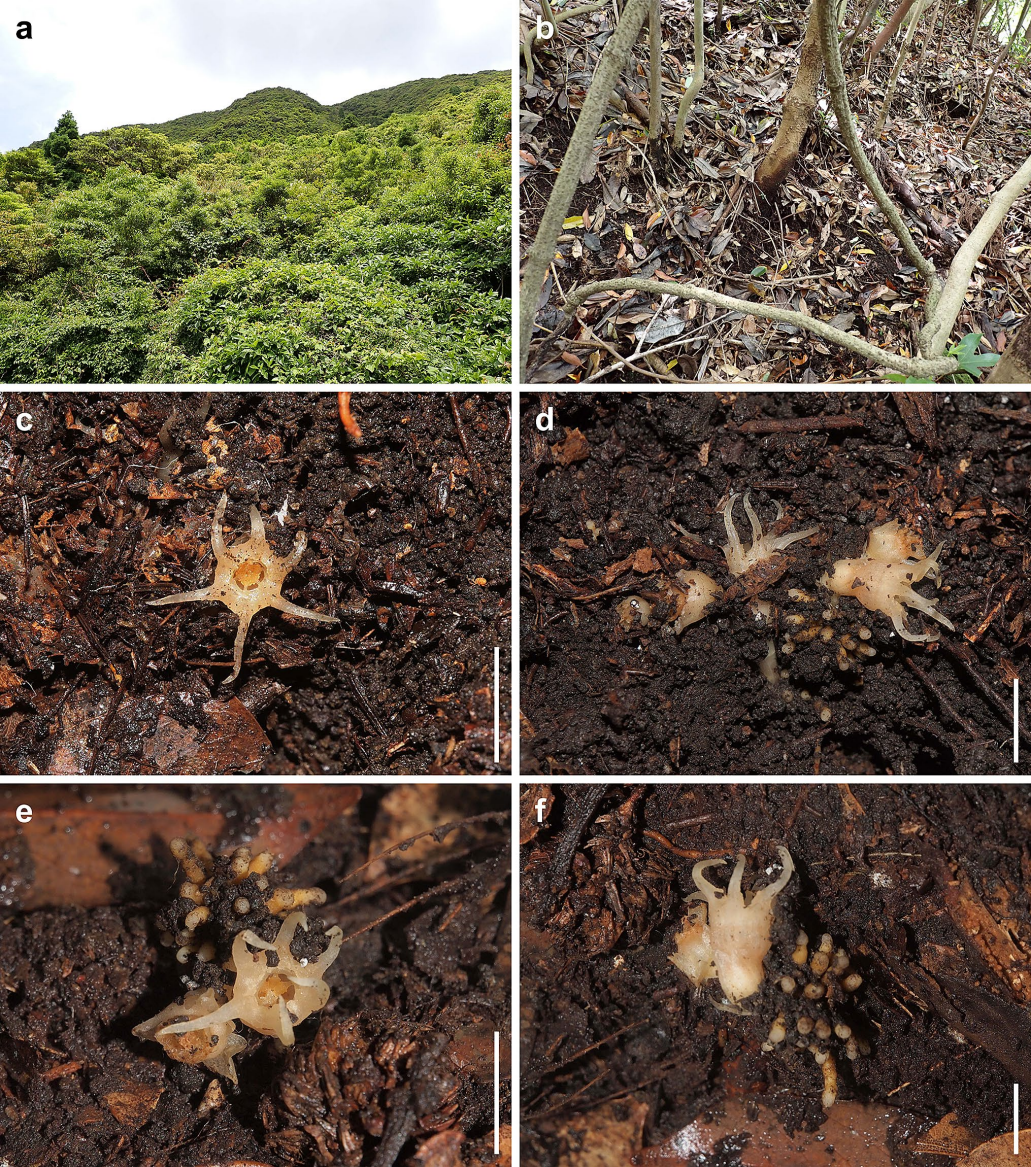
*Relictithismia kimotsukiensis* and its habitat at the type locality. **a** & **b** A secondary evergreen broad-leaved forest where *R. kimotsukiensis* was found. **c** & **e** Flower, top view. **d** & **f** Flower, lateral view. Scale bars: 10 mm. Photos (**a** & **b**) were taken by Yasunori Nakamura on 3 June 2022, and photos (**c**–**f**) were taken by Shuichiro Tagane on 9 June 2023. Photos (**e** & **f**) were captured after collection

During a floristic survey on Osumi Peninsula, Kagoshima Prefecture, Kyushu Island, southern Japan in 2022, the second author YN collected an unknown plant belonging to the family Thismiaceae in a secondary evergreen broad-leaved forest (Fig. 1). It differed from the species of the two genera of Thismiaceae, *Oxygyne* and *Thismia*, distributed in East Asia, in having six stamens (vs. three in *Oxygyne*) and free stamens touching the stigma during anthesis, a trait absent in *Thismia* (Cheek et al. 2018; Nuraliev et al. 2021; Shepeleva et al. 2020). The characteristic of free stamens touching the stigma is reminiscent of *Afrothismia*, a genus exclusively found in Africa (Cheek et al. 2023). However, it differs from *Afrothismia* in having tuberous roots, an actinomorphic flower, and a non-segmented tube. So far, a cluster of tuberous roots has been known only in the monotypic genus *Haplothismia* found in India. However, the unknown plant also differs from *H. exannulata* Airy Shaw in having one or two solitary flowers, free thecae, and the presence of an annulus (Airy-Shaw 1952; Sasidharan and Sujanapal 2000; Shepeleva et al. 2020). Furthermore, our phylogenetic analysis showed that the unknown plant exhibits significant genetic divergence from members of both Old World Thismiaceae (*Haplothismia*, *Oxygyne*, and *Thismia*, subg. *Thismia*) and New World Thismiaceae (*Thismia* subg. *Ophiomeris* and *Tiputinia*), suggesting it represents a distinct lineage.

Consequently, we propose a new genus, *Relictithismia* Suetsugu & Tagane, along with the newly found species *Relictithismia kimotsukiensis* Suetsugu, Yas.Nakam. & Tagane. This paper includes a detailed description, illustrations, preliminary conservation status, notes on the phylogenetic relationship, evolutionary history, and conservation assessment of *R. kimotsukiensis*.

## Materials and methods

### Morphological observations

An individual of *Relictithismia kimotsukiensis* was collected from Kimotsuki-cho, Kagoshima Prefecture (its type locality) on 3 June 2022, and three additional *R. kimotsukiensis* individuals were also collected at the same locality on 9 June 2023. Given that precise identification of Thismiaceae species often hinges on the analysis of floral characteristics within the floral tube, their flowers were carefully dissected to examine their inner floral morphology. Morphological characteristics were observed under a Leica M165C and MZ95 stereomicroscope and measured using a digital caliper. Additionally, the dissected flowering specimens were photographed with a Leica MC170 HD digital camera attached to the same microscope or an OLYMPUS TG-3.

To investigate the morphological characteristics of other Thismiaceae species, we also reviewed relevant literature and online digitized plant collections, including JSTOR Global Plants (http://plants.jstor.org/). Furthermore, we examined herbarium specimens deposited at FOF, HYO, K, KAG, KYO, RYU, TI, TNS, and VNM. These herbarium acronyms follow the Index Herbariorum (Thiers 2023).

### DNA extraction, PCR amplification, and sequencing

Genomic DNA was extracted from the scale leaves of two *Relictithismia kimotsukiensis* individuals collected on 9 June 2023, using the CTAB method (Doyle and Doyle 1990). The primer sets used for the PCR and cycle sequencing (CS) reactions in this study were as follows: for 18S rDNA (18S), NS1 and NS2 (PCR and CS), NS3 and NS4 (PCR and CS), as well as NS5 and NS8 (PCR and CS) (White et al. 1990) and for ATP synthase subunit A (*atpA*), ATPA-F1 and ATPA-B1 (PCR and CS) (Eyre-Walker and Gaut 1997), with ATPA-F5 and ATPA-B5 (CS only) (Davis et al. 1998). The PCR reaction and DNA sequencing were performed using the modified methods mentioned in Nakano (2012), with the aid of a T100 Thermal Cycler (Bio-Rad, Hercules, USA).

The PCR reactions were initiated with an initial heating step at 95 °C for 5 min, followed by 35 cycles of amplification. For the 18S primer sets, each cycle involved three stages: denaturation at 94 °C for 10 s, annealing at 52 °C for 20 s, and extension at 72 °C for 42 s. For the *atpA* primer set, the cycles included stages at 94 °C for 10 s, annealing at 54 °C for 20 s, and extension at 72 °C for 73 s. Subsequently, the sequencing mixtures were subjected to an initial heating step at 96 °C for 2 min, followed by 40 cycles of sequencing, with each cycle consisting of denaturation at 96 °C for 10 s, annealing at 50 °C for 5 s, and extension at 60 °C for 48 s. The obtained sequences were edited using DNA BASER (Heracle Biosoft S.R.L., Piteşti, Romania). The four DNA sequences newly obtained in this study have been deposited with the International Nucleotide Sequence Databases (INSD) through the DNA Data Bank of Japan (Table S1).

### Sequence alignment and phylogenetic analysis

In addition to the newly obtained sequences of *Relictithismia kimotsukiensis*, 21 sequences representing nine species of Thismiaceae, and two species of *Tacca* (outgroup) were retrieved from the INSD based on the selection criteria following Shepeleva et al. (2020), and were included in the present dataset (Table S1). Given the fact that the respective 18S and *atpA* sequences of the two *R. kimotsukiensis* individuals were completely identical to each other, the duplicate sequences were not included in the present dataset. The sequences of 18S were aligned using MAFFT Server v. 7.511 Q-INS-i (Katoh and Toh 2008; Katoh and Standley 2013; Kuraku et al. 2013) with the McCaskill routine from the Vienna RNA package (Hofacker et al. 2002) and MXSCARNA (Tabei et al. 2008), while the sequences of *atpA* were aligned using MAFFT v. 7.520 L-INS-i. The aligned 18S and *atpA* sequences were of lengths 1758 and 1275 bp, respectively, resulting in a concatenated alignment of 3033 bp.

Phylogenetic trees were reconstructed using maximum likelihood (ML) and Bayesian inference (BI). The best-fit partition scheme and models were identified based on the Bayesian information criterion using ModelFinder (Kalyaanamoorthy et al. 2017) implemented in IQ-Tree v. 2.2.2.6 (Minh et al. 2020). The selected partition scheme and models for each genetic marker were as follows: for 18S, GTR + I + G; for *atpA* 1st position, GTR + I; and for *atpA* 2nd and 3rd positions, F81 + I. The ML tree was calculated using IQ-Tree v. 2.2.2.6 with non-parametric bootstrapping (BS) conducted with 1000 replicates. The BI tree and Bayesian posterior probabilities (PP) were estimated using MrBayes v. 3.2.7a (Ronquist et al. 2012). Two independent runs for four Markov chains were conducted for 4 million generations, and the BI tree was sampled every 100 generations. The parameter estimates and convergence were checked using Tracer v. 1.7.1 (Rambaut et al. 2018), and then the first 10,001 trees were discarded based on the results.

## Results and discussion

### Morphological comparison of *Relictithismia* and other Thismiaceae genera

The thorough review and examination of herbarium specimens, protologues, and living plants revealed a distinct set of morphological characteristics that consistently distinguish *Relictithismia* from the previously known five genera of Thismiaceae *s.l.* (*Afrothismia*, *Haplothismia*, *Oxygyne*, *Thismia*, and *Tiputinia*).

**Fig. 2:**
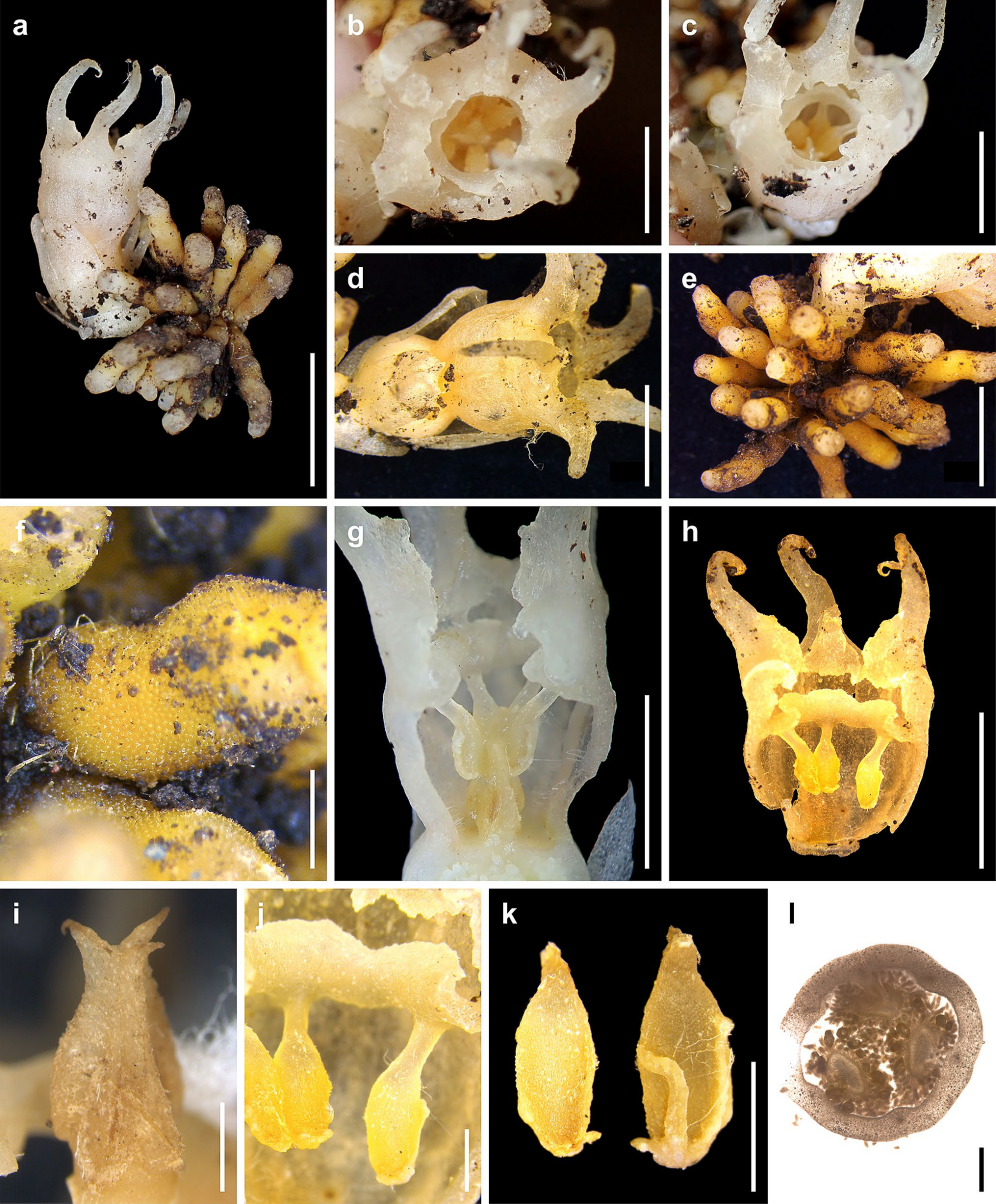
*Relictithismia kimotsukiensis*. **a** Habit. **b** Flower, top view. **c** Flower, diagonal top view. **d** Flower, lateral view. **e** Tuberous roots. **f** Close-up of root. **g** Longitudinal section of floral tube showing stamens, style, and stigma. **h** Longitudinal section of floral tube with stamens. **i** Style and stigma. **j** Annulus and stamens. **k** Anthers. **l** Transverse section of ovary with three equal placentas and numerous ovules. Materials from *Y. Nakamura & S. Tagane 23060901* (**a**–**f**, **h**–**l** for TNS, **g** for KAG). Scale bars: 10 mm (**a**), 5 mm (**b**–**e** and **g**–**h**), and 1 mm (**f** and **i**–**l**)

**Fig. 3:**
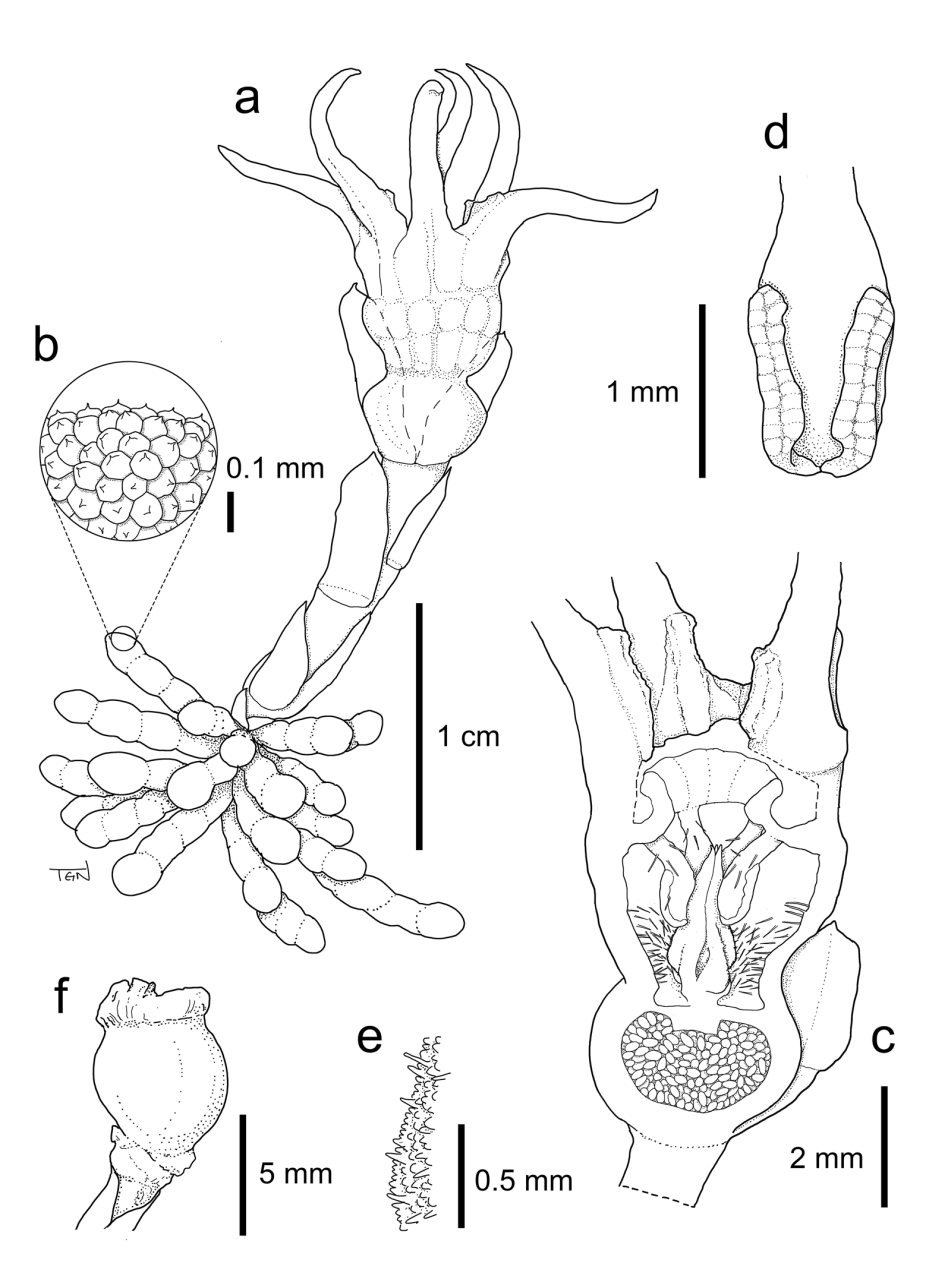
*Relictithismia kimotsukiensis*. **a** Habit. **b** Close-up of root surface. **c** Longitudinal section of flower. **d** Anther. **e** Stigma surface.** f** Immature fruit. Materials from *Y. Nakamura & S. Tagane 23060901* (KAG). Drawn by S. Tagane

In East Asia, two genera *Oxygyne* and *Thismia* within Thismiaceae have been known, both of which occur in Japan (Cheek et al. 2018). Upon detailed examination, we concluded that *R. kimotsukiensis* did not correspond to either *Oxygyne* or *Thismia*. *Relictithismia* is characterized by possessing six stamens (Fig. 2), setting it apart from *Oxygyne*, which has three stamens (Cheek et al. 2018; Shepeleva et al. 2020; Suetsugu et al. 2019). Notably, its stamens are free from each other, pendulous, and touch the stigma during anthesis (Figs. 2g, 3c). This is in stark contrast to *Thismia*, which is known for stamens with dilated connectives, forming a staminal tube (except in the paraphyletic Neotropical subgenus *Ophiomeris* (Miers) Maas & H.Maas), and never touching the stigma (Nuraliev et al. 2021; Shepeleva et al. 2020). It is also noteworthy that although some members of the Neotropical subgenus *Ophiomeris* also feature stamens without dilated connectives, *Relictithismia* differs from *Ophiomeris* in having a cluster of tuberous roots (vs. a single globose tuber with numerous filiform roots or vermiform roots in *Ophiomeris*) and the annulus located inside the perianth mouth (vs. the annulus located at the mouth of the floral tube) (Maas et al. 1986; Shepeleva et al. 2020).

*Relictithismia* resembles the monotypic genus *Haplothismia*, known from India, in having a cluster of tuberous roots and homomorphic outer and inner perianth lobes with long filiform appendages (Figs. 1, 2 and 3). Notably, so far, a cluster of tuberous roots has been known only in *Haplothismia* within the Thismiaceae (Airy-Shaw 1952; Sasidharan and Sujanapal 2000; Shepeleva et al. 2020). Several recent studies have suggested that, although the outer floral appearance may have little phylogenetic significance in Thismiaceae classification possibly due to convergent evolution based on pollinator preferences (Shepeleva et al. 2020), the structure of the underground organs is a stable trait reflecting phylogenetic information (Feller et al. 2022; Imhof et al. 2020). Thus, *R. kimotsukiensis* might be most closely related to *Haplothismia.* However, it can be distinguished from *Haplothismia* by the following several key features: solitary flowers (vs. 2–6-flowered pseudo-raceme in *Haplothismia*), largely separate anther thecae (vs. connate), the presence of an annulus (vs. absent), and stamens touching the stigma (vs. apart from the stigma) (Airy-Shaw 1952; Sasidharan and Sujanapal 2000; Shepeleva et al. 2020).

The anther touching the stigma on *R. kimotsukiensis* is reminiscent of the African genus *Afrothismia*, as this feature was previously observed only in this genus within Thismiaceae *s.l.* (Cheek et al. 2023)*.* However, in contrast to *Afrothismia*, which features an underground system composed of short rhizome bearing clusters of globose bulbils, each with a terminal rootlet, a zygomorphic flower, and a two-chambered floral tube (Cheek et al. 2023), our specimen exhibits a cluster of tuberous roots, an actinomorphic flower, and a non-segmented floral tube. Additionally, not only *Relictithismia* but also *Haplothismia*, *Oxygyne*, *Thismia*, and *Tiputinia* differ from *Afrothismia* in having the ovary with three placentas (vs. ovary with a single placenta) (Fig. 2l). Given that *Afrothismia* is not monophyletic with other Thismiaceae species (Lin et al. 2022; Merckx et al. 2009), the free stamens touching the stigma likely represent an example of convergent evolution for facilitating self-pollination. Notably, autonomous self-pollination is advantageous for mycoheterotrophic plants in dark, shaded forest understories where pollinators are scarce (Suetsugu 2013, 2015, 2022). Consequently, these morphological traits can be regarded as mechanisms providing reproductive assurance.

Finally, *Relictithismia* also differs from the monotypic genus *Tiputinia*, found in Ecuador of South America in having a cluster of tuberous roots (vs. vertical cylindric sympodially branched rhizome in *Tiputinia*), stamens without filament appendages and interstaminal lobes (vs. stamens with five or six pairs of filament appendages and two globoid interstaminal lobes), the presence of an annulus (vs. absent), and stamens touching the stigma (vs. free, never touching the stigma) (Woodward et al. 2007). Overall, these morphological data indicated that our specimen represents an isolated lineage under the family Thismiaceae.

### Phylogenetic comparison of *Relictithismia* and other Thismiaceae genera

**Fig. 4:**
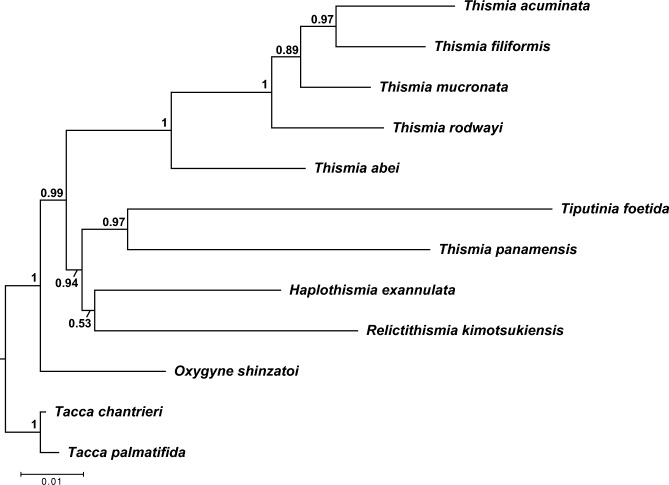
Bayesian inference phylogenetic tree of the combined 18S rDNA and *atpA* sequences from *Relictithismia kimotsukiensis* and its related taxa. Node values indicate posterior probability values. The scale bar represents the number of substitutions per site

Both the BI (Fig. 4) and ML (Fig. S1) trees exhibited nearly identical topologies, largely consistent with the findings of Shepeleva et al. (2020). The monophyly of the Thismiaceae received strong support in both analyses (BS = 100%, PP = 1.0).

Echoing recent studies (Merckx et al. 2006, 2009; Merckx and Smets 2014; Shepeleva et al. 2020; Yokoyama et al. 2008), our findings confirm that *Thismia* is polyphyletic. Currently, *Thismia* is split into two subgenera: the subgenus *Thismia*, which includes all Old World species and *T. americana,* and *Ophiomeris*, which encompasses Neotropical species (Kumar et al. 2017; Maas et al. 1986; Merckx and Smets 2014). The Old World *Thismia* clade, which aligns with the subgenus *Thismia*, was confidently retrieved (BS = 100%, PP = 1.0), while the subgenus *Ophiomeris*, represented by *Thismia panamensis* (Standl.) Jonker of the New World, is distantly related to the subgenus *Thismia.* Given the morphological distinction where *Ophiomeris* stamens are free from each other, in contrast to the connate or connivent stamens forming a staminal tube in the subgenus *Thismia* (Shepeleva et al. 2020; Yang et al. 2010), these two are differentiated based on both morphological and molecular evidence. It might be reasonable to classify *Ophiomeris* at the genus level. However, considering the morphological variability of Neotropical *Thismia* (Maas et al. 1986), there is a question as to whether all of them are closely related, forming a single monophyletic genus. Regrettably, molecular datasets for other *Ophiomeris* species are currently unavailable. Therefore, acquiring molecular data from additional *Ophiomeris* species is crucial to substantiate their phylogenetic relationships.

Our findings also indicated that *Th. panamensis*, *Tiputinia foetida* P.E.Berry & C.L.Woodw., *Haplothismia exannulata*, and *Relictithismia kimotsukiensis* occupy an early-diverging position in this family, positioned outside the Old World *Thismia* clade (the subgenus *Thismia*). Notably, these species have less specialized perianths than those of the subgenus *Thismia*, possessing identical inner and outer perianth whorls and free stamens (Airy-Shaw 1952; Cheek et al. 2018; Shepeleva et al. 2020; Woodward et al. 2007). *Thismia panamensis*, *Ti. foetida*, *H. exannulata*, and *R. kimotsukiensis* formed a relatively well-supported monophyletic lineage in the BI analysis (PP = 0.94), while this clade did not receive support in the ML analysis (BS = 48%). Additionally, the current phylogenies were unable to resolve robust relationships among these four species, although the monophyly of *Th. panamensis* and *Ti. foetida* was well-supported in both analyses (BS = 84%, PP = 0.96).

The cluster of tuberous roots of *R. kimotsukiensis* suggests some affinity with *Haplothismia*. On the other hand, the presence of pendulous stamens from the annulus can be found only in *R. kimotsukiensis* and *Thismia.* Thus, *Relictithismia* could potentially act as a connecting link between other early-diverging genera in Thismiaceae (particularly *Haplothismia*) and *Thismia.* However, the precise phylogenetic relationships among *Relictithismia*, *Haplothismia*, *Ophiomeris*, and *Tiputinia* remain uncertain. It is crucial to employ high-throughput sequencing technology for a more detailed understanding of their phylogenetic relationships and morphological evolution. Even if a monophyletic lineage between *H. exannulata* and *R. kimotsukiensis* is confirmed, it is noteworthy that sufficient genetic divergence between these two taxa shown in our phylogenetic analysis is comparable to that between other genera.

Overall, the available phylogenetic data, along with the morphological distinctness, strongly support the conclusion that *Relictithismia kimotsukiensis* does not align with any existing genera. Therefore, we propose a new genus for this taxon, which is described in detail in the following sections.

### Taxonomic treatments

***Relictithismia*** Suetsugu & Tagane, gen. nov.

**Diagnosis** *Relictithismia* resembles *Haplothismia* in possessing a cluster of tuberous roots, yet it is clearly distinguished by its solitary flowers (vs. 2–6-flowered pseudo-raceme in *Haplothismia*), largely separate anther thecae (vs. connate), and the presence of an annulus (vs. absence).

**Type species** *Relictithismia kimotsukiensis* Suetsugu, Yas.Nakam. & Tagane.

**Description** Achlorophyllous herbs. Underground part consisting of a short rhizomatous stem and a cluster of tuberous roots. Stems 1–2 per plant, endogenous, terminating aboveground in a single flower. Leaves spirally arranged, simple, sessile, reduced to scales, appressed on the stem. Floral bracts usually 3 in one whorl, not equal in size, erect, subtending the flower, ovate-triangular to ovate-oblong. Flower solitary, actinomorphic, epigynous; floral tube obpyriform lacking distinctive ridges; perianth lobes 6, equal, patent or ascending, filiform to lanceolate with a callus-like cluster of round cells on the adaxial surface at base; annulus present, inside the floral tube. Stamens 6, pendulous from the annulus, arching inward and touching the stigma; filaments slightly dorsiventrally flattened, adorned with some long hairs; anther thecae 2, largely separated, elliptic, dehiscing longitudinally towards the abaxial side. Ovary inferior, 1-locular with 3 parietal placentae; ovules numerous; style cylindrical; stigma conical, apically 3-lobed.

**Etymology** The name of the new genus, *Relictithismia*, is a combination of the Latin word “relictus”, meaning “left behind”, and the generic name *Thismia*. The name reflects its morphological characteristics that serve as a bridge between the early-diverging genera in Thismiaceae and the genus *Thismia*, showcasing the putative ancestral condition of these plant groups.

***Relictithismia kimotsukiensis*** Suetsugu, Yas.Nakam. & Tagane, sp. nov. (Figs. 1–3).

**Type** JAPAN. Kagoshima Prefecture, Kimotsuki-gun, Kimotsuki-cho, 9 June 2023, *Y. Nakamura & S. Tagane 23060901* (holotype TNS [TNS VS-8001405 in spirit!], isotypes KAG [KAG183094!], KYO [KYO00027080 in spirit!]).

**Description** Achlorophyllous herbs. Roots clustered, radiating from the stem base, up to 28, 5.3–20.0 mm long, 1.3–2.2 mm in diam., light brown, tuberous, warty. Rhizome not elongated. Stems 1–2 per plant, white, simple, erect, 5–16 mm long at anthesis, elongated to 21 mm long at immature fruiting stage. Leaves scale-like, spirally arranged, 8–11, appressed on the stem, ovate-triangular to narrowly ovate-oblong, 1.5–7.8 × 1.3–3.8 mm, white, apex acute to obtuse. Floral bracts 3(–4) in one whorl, not equal in size, erect, subtending the flower, ovate-triangular to ovate-oblong, 5.1–8.9 × 3.2–4.5 mm, white, apex obtuse. Flower white, solitary, actinomorphic, epigynous. Perianth basally fused, forming an obpyriform floral tube with 6 free perianth lobes. Floral tube 10.5–12.0 mm long, 6.2–7.0 mm in diam., outer surface glabrous, inner surface without transverse bars, densely hairy at lower 2/3, with an annulus 1.9–2.1 mm below the mouth; mouth circular, 3.4–4.8 mm in diam. Perianth lobes 6, equal, filiform to lanceolate, 9–12 mm long, slightly recurved near apex, with a callus-like cluster of round cells on adaxial surface at base; margins of adjacent lobes fused. Stamens 6, free, pendulous from the annulus, arching inward and touching the stigma; filaments slightly dorsiventrally flattened, 0.6–1.0 mm long, sparsely covered with long hairs; anthers 1.5–1.7 × 0.8–1 mm long, slightly papillate, with some long hairs, anther thecae 2, largely separated, oblong, 1.0–1.3 mm in length, dehiscing longitudinally to the abaxial side. Ovary depressed globose, 3.0–3.8 mm long, 3.5–4.6 mm in diam., 1-locular with 3 parietal placentae, ovules numerous. Style cylindrical, 0.3–0.5 mm long, stigma conical, 2.6–2.7 × 1.5–1.7 mm, 3-ridged, densely covered with papillate to subulate trichomes, apically 3-lobed. Immature fruits slightly depressed globose, 5.6–5.8 mm long, 6.1–6.6 mm in diam. Seeds not seen.

**Additional specimen examined (paratype)** JAPAN. Kagoshima Prefecture, Kimotsuki-gun, Kimotsuki-cho, 3 June 2022, *Y. Nakamura 22060301* (KAG [KAG180387-in spirit!])*.*

**Etymology** The species epithet refers to the type locality of the new species.

**Vernacular name** Mujina-no-shokudai (in Japanese). The vernacular name is inspired by its apparent resemblance to and the distinct diffrences from species of *Thismia*, locally referred to as “tanuki-no-shokudai,” meaning a candlestick used by a raccoon dog. The term “mujina” is an old Japanese name for a badger, known as “anaguma” in Japanese, although historically and regionally, it has sometimes also been associated with raccoon dogs. Therefore, we consider that this name aptly represents the similarity and differences between *Thismia* and *Relictithismia*. Moreover, the name “mujina-no-shokudai” (= a candlestick used by a badger) fits its ecology, as the majority of the plant, even during the flowering stage, remains buried underground beneath fallen leaves, reminiscent of a badger burrowing underground.

**Distribution** Japan, Kagoshima Pref., Kimotsuki-gun, Kimotsuki-cho (currently known only from the type locality).

**Habitat** The species occurs in the understory of a secondary evergreen broad-leaved forest mixed with a few planted *Cryptomeria japonica* (L.f.) D.Don (Cupressaceae). The forest is dominated by *Ilex integra* Thunb. (Aquifoliaceae), *Dendropanax trifidus* (Thunb.) Makino ex H.Hara (Araliaceae), *Daphniphyllum macropodum* Miq. subsp. *macropodum* (Daphniphyllaceae), *Callicarpa japonica* Thunb. var. *luxurians* Rehder (Lamiaceae), *Cinnamomum yabunikkei* H.Ohba, *Machilus japonicus* Siebold et Zucc. ex Blume, *Machilus thunbergii* Siebold et Zucc., *Neolitsea sericea* (Blume) Koidz. (Lauraceae), *Ficus erecta* Thunb. (Moraceae), *Ligustrum japonicum* Thunb. (Oleaceae), *Illicium anisatum* L. (Schisandraceae), *Symplocos kuroki* Nagam. (Symplocaceae), and *Eurya japonica* Thunb. (Pentaphylacaceae). In the understory, some shrubs and herbs, including *Arachniodes sporadosora* (Kunze) Nakaike (Dryopteridaceae), *Epipogium roseum* (D.Don) Lindl., *Goodyera similis* Blume (Orchidaceae), *Maesa japonica* (Thunb.) Moritzi et Zoll. (Primulaceae), and *Rubus buergeri* Miq. (Rosaceae), are scattered.

**Phenology** Flowers and immature fruits were observed and collected in early June.

**INSD accession no.** LC775771 & LC775772 (18S), LC775773 & LC775774 (*aptA*); obtained from *Y. Nakamura & S. Tagane 23060901* (holotype in TNS & isotype in KYO, respectively).

**Preliminary conservation assessment** Critically Endangered (CR). We assess *Relictithismia kimotsukiensis* as “Critically Endangered” [B1 & 2a, b(iii) + C2a(i, ii) + D] following the IUCN Red List Categories and Criteria (IUCN 2022). This species is known only from a single location, where the above specimens were collected and fewer than five individuals were observed in total. Initially, it was discovered by YN with a single specimen on 3 June 2022, but subsequent field surveys in early June (by ST and KS) and late June (by YN) of the same year at the type locality did not yield additional findings. However, a reassessment on 9 June 2023 (by YN and ST), resulted in the discovery of four more individuals. The locality, adjacent to a forest road and outside a protected area, features mostly young trees with a diameter at breast height (DBH) of less than 13 cm, indicating recent forest clearing within the past 20–30 years. Despite extensive searches by YN, ST, and KS around the population during the flowering season, this species has not been found in any other areas.

Given its extremely limited distribution and potential habitat threats, *R. kimotsukiensis* is classified as Critically Endangered. However, due to its cryptic growth habit and ephemeral flowering phenology, *R. kimotsukiensis* may exist in other, yet undiscovered locations. Therefore, further intensive field surveys and subsequent observations are essential to more fully comprehend its distribution and elusive natural history.

**Possible pollination mode** *Relictithismia kimotsukiensis* exhibits floral traits suggestive of sapromyophily, a pollination strategy that involves attracting fly pollinators. Traits such as the filiform appendages of the perianth lobes in *R. kimotsukiensis* are largely aligned with adaptations for attracting fly pollinators (Suetsugu et al. 2022a). Notably, dipteran pollinators have been observed being temporarily restrained inside the floral tube of some Thismiaceae species (Guo et al. 2019; Suetsugu and Sueyoshi 2021).

Nonetheless, the floral structure of *R. kimotsukiensis*, particularly the stamens reaching the stigma surface at anthesis, implies an adaptation for self-pollination. Overall, we consider self-pollination to be a plausible predominant mode in *R. kimotsukiensis*, although the possibility of some contribution from insect-mediated pollination cannot be completely ruled out.

**Biogeographical note** Japan boasts the highest diversity of genera within the family Thismiaceae, hosting three distinct genera. Intriguingly, the genus *Oxygyne*, the probable earliest-diverging member of the Thismiaceae, coexists in Japan with both *Thismia* and *Relictithismia*. As discussed above, *Relictithismia* possesses morphological features that bridge the early-diverging genera in Thismiaceae and the genus *Thismia*. It is also noteworthy that the two Japanese *Thismia* species, *T. abei* and *T. kobensis*, are early-diverging members within the Old World *Thismia* clade (Shepeleva et al. 2020; Suetsugu et al. 2023). Detailed studies on Japanese Thismiaceae species are likely to provide crucial insights into the biogeography and evolutionary history of the Thismiaceae as a whole.

### Key to the genus of Thismiaceae *s.l.* (modified from Cheek et al. 2018)

1a. Staminal filaments inserted at the mouth of the floral tube, erect and exserted at base, then arching inwards, the anthers held at the level of the tube mouth, or just inside…………. 2

1b. Staminal filaments inserted inside the floral tube, or in the throat of the floral tube,, the anthers held inside the tube…………. 4

2a. Stamens 6, annulus absent…………. 3

2b. Stamens 3, annulus lamellae present…………. ***Oxygyne***

3a. Inflorescences aerial, with multiple branches and flowers; stamens without filament appendages and interstaminal lobes…………. ***Haplothismia***

3b. Inflorescences at ground level, usually 1-flowered; stamens with five or six pairs of filament appendages and two globoid interstaminal lobes…………. ***Tiputinia***

4a. Annulus located inside the floral tube; stamens inserted in the lower floral tube where they are attached to the stigma…………. 5

4b. Annulus located at the mouth of floral tube; stamens free of and distant from the stigma…………. ***Thismia***

5a. Staminal filaments adjacent to annulus; underground system with tuberous roots…………. ***Relictithismia***

5b. Stamens inserted below the annulus; underground system with rhizome bearing clusters of globose bulbils, each with a terminal rootlet…………. ***Afrothismia***

### Supplementary Information

Below is the link to the electronic supplementary material.

Supplementary file1 (PDF 133 KB)

## Data Availability

The sequences obtained have been deposited in the DDBJ/ENA/GenBank databases under accession numbers LC775771–LC775774. Additional supporting information is available online in the Supporting Information section at the end of the article.

## References

[CR1] Airy-Shaw HK (1952). A new genus and species of Burmanniaceae from South India. Kew Bull.

[CR2] Cheek M, Tsukaya H, Rudall PJ, Suetsugu K (2018). Taxonomic monograph of *Oxygyne* (Thismiaceae), rare achlorophyllous mycoheterotrophs with strongly disjunct distribution. PeerJ.

[CR3] Cheek M, Gomez MS, Graham SW, Rudall PJ (2023). Afrothismiaceae (Dioscoreales), a new fully mycoheterotrophic family endemic to tropical Africa. Kew Bull.

[CR4] Dančák M, Hroneš M, Sochor M (2020). *Thismia*: the rarest of the rare? Ranges of some Bornean species are much larger than previously believed. Phytotaxa.

[CR5] Davis JI, Simmons MP, Stevenson DW, Wendel JF (1998). Data decisiveness, data quality, and incongruence in phylogenetic analysis: an example from the monocotyledons using mitochondrial *atp* A sequences. Syst Biol.

[CR6] Doyle JJ, Doyle JL (1990). Isolation of plant DNA from fresh tissue. Focus.

[CR7] Eyre-Walker A, Gaut BS (1997). Correlated rates of synonymous site evolution across plant genomes. Mol Biol Evol.

[CR8] Feller B, Dančák M, Hroneš M (2022). Mycorrhizal structures in mycoheterotrophic *Thismia* spp. (Thismiaceae): functional and evolutionary interpretations. Mycorrhiza.

[CR9] Garrett N, Viruel J, Klimpert N (2023). Plastid phylogenomics and molecular evolution of Thismiaceae (Dioscoreales). Am J Bot.

[CR10] Gomes SIF, Aguirre-Gutierrez J, Bidartondo MI, Merckx V (2017). Arbuscular mycorrhizal interactions of mycoheterotrophic *Thismia* are more specialized than in autotrophic plants. New Phytol.

[CR11] Guo X, Zhao Z, Mar SS (2019). A symbiotic balancing act: arbuscular mycorrhizal specificity and specialist fungus gnat pollination in the mycoheterotrophic genus *Thismia* (Thismiaceae). Ann Bot.

[CR12] Hofacker IL, Fekete M, Stadler PF (2002). Secondary structure prediction for aligned RNA sequences. J Mol Biol.

[CR13] Imhof S, Feller B, Heser A (2020). Morpho-anatomical differences among mycoheterotrophic *Afrothismia* spp. (Thismiaceae) indicate an evolutionary progression towards improved mycorrhizal benefit. Mycorrhiza.

[CR14] IUCN (2022) Guidelines for using the IUCN Red List Categories and Criteria. Version 15.1. Prepared by the Standards and Petitions Committee of the IUCN Species Survival Commission. https://www.iucnredlist.org/documents/RedListGuidelines.pdf. Accessed 8 Dec 2023

[CR15] Kalyaanamoorthy S, Minh BQ, Wong TKF (2017). ModelFinder: fast model selection for accurate phylogenetic estimates. Nat Methods.

[CR16] Katoh K, Standley DM (2013). MAFFT multiple sequence alignment software version 7: improvements in performance and usability. Mol Biol Evol.

[CR17] Katoh K, Toh H (2008). Improved accuracy of multiple ncRNA alignment by incorporating structural information into a MAFFT-based framework. BMC Bioinformatics.

[CR18] Kubota Y, Kusumoto B, Shiono T, Tanaka T (2017). Phylogenetic properties of Tertiary relict flora in the east Asian continental islands: imprint of climatic niche conservatism and in situ diversification. Ecography.

[CR19] Kumar P, Gale SW, Li J-H (2017). *Thismia nigricoronata*, a new species of Burmanniaceae (Thismieae, Dioscoreales) from Vang Vieng, Vientiane Province, Laos, and a key to subgeneric classification. Phytotaxa.

[CR20] Kuraku S, Zmasek CM, Nishimura O, Katoh K (2013). aLeaves facilitates on-demand exploration of metazoan gene family trees on MAFFT sequence alignment server with enhanced interactivity. Nucleic Acids Res.

[CR21] Lam VKY, Darby H, Merckx VSFT (2018). Phylogenomic inference in extremis: a case study with mycoheterotroph plastomes. Am J Bot.

[CR22] Lam VKY (2016) Phylogenomics and comparative plastome analysis of mycoheterotrophic plants. Dissertation, University of British Columbia. 10.14288/1.0305829

[CR23] Lin Q, Braukmann TWA, Soto Gomez M (2022). Mitochondrial genomic data are effective at placing mycoheterotrophic lineages in plant phylogeny. New Phytol.

[CR24] Maas PJM, Maas-van de Kamer H, van Benthem J (1986). Burmanniaceae. Flora Neotropica Monogr.

[CR25] Merckx VSFT, Smets EF (2014). *Thismia americana*, the 101st anniversary of a botanical mystery. Int J Plant Sci.

[CR26] Merckx V, Schols P, Kamer HM (2006). Phylogeny and evolution of Burmanniaceae (Dioscoreales) based on nuclear and mitochondrial data. Am J Bot.

[CR27] Merckx V, Bakker FT, Huysmans S, Smets E (2009). Bias and conflict in phylogenetic inference of myco-heterotrophic plants: a case study in Thismiaceae. Cladistics.

[CR28] Millien-Parra V, Jaeger J-J (1999). Island biogeography of the Japanese terrestrial mammal assemblages: an example of a relict fauna. J Biogeogr.

[CR29] Milne RI, Abbott RJ (2002). The origin and evolution of tertiary relict floras. Adv Bot Res.

[CR30] Minh BQ, Schmidt HA, Chernomor O (2020). IQ-TREE 2: new models and efficient methods for phylogenetic inference in the genomic era. Mol Biol Evol.

[CR31] Nakano T (2012). A new species of *Orobdella* (Hirudinida, Arhynchobdellida, Gastrostomobdellidae) and redescription of *O. kawakatsuorum* from Hokkaido, Japan with the phylogenetic position of the new species. ZooKeys.

[CR32] Nuraliev MS, Yudina SV, Shepeleva EA (2021). Floral structure in *Thismia* (Thismiaceae: Dioscoreales): new insights from anatomy, vasculature and development. Bot J Linn Soc.

[CR33] POWO (2024) Plants of the World Online. Royal Botanic Gardens, Kew. http://www.plantsoftheworldonline.org/. Accessed 12 Jan 2024

[CR34] Rambaut A, Drummond AJ, Xie D (2018). Posterior summarization in Bayesian phylogenetics using Tracer 1.7. Syst Biol.

[CR35] Ronquist F, Teslenko M, van der Mark P (2012). MrBayes 3.2: efficient Bayesian phylogenetic inference and model choice across a large model space. Syst Biol.

[CR36] Sasidharan N, Sujanapal P (2000). Rediscovery of *Haplothismia exannulata* Airy Shaw (Burmanniaceae) from its type locality. Rheedea.

[CR37] Shepeleva EA, Schelkunov MI, Hroneš M (2020). Phylogenetics of the mycoheterotrophic genus *Thismia* (Thismiaceae: Dioscoreales) with a focus on the Old World taxa: delineation of novel natural groups and insights into the evolution of morphological traits. Bot J Linn Soc.

[CR38] Suetsugu K (2013). Autogamous fruit set in a mycoheterotrophic orchid * Cyrtosia septentrionalis*. Plant Syst Evol.

[CR39] Suetsugu K (2015). Autonomous self-pollination and insect visitors in partially and fully mycoheterotrophic species of *Cymbidium* (Orchidaceae). J Plant Res.

[CR40] Suetsugu K (2022). Living in the shadows: *Gastrodia* orchids lack functional leaves and open flowers. Plants People Planet.

[CR41] Suetsugu K, Nishioka T (2017). *Sciaphila sugimotoi* (Triuridaceae), a new mycoheterotrophic plant from Ishigaki Island, Japan. Phytotaxa.

[CR42] Suetsugu K, Sueyoshi M (2021). Fairy lanterns may lure pollinators by mimicking fungi. Front Ecol Environ.

[CR43] Suetsugu K, Sugimoto T, Tsukaya H (2019). Emended description and new localities of *Oxygyne shinzatoi* (Burmanniaceae/Thismiaceae), with discussion of phylogenetic relationships of *Oxygyne* from Japan and Africa. Phytotaxa.

[CR44] Suetsugu K, Nishigaki H, Fukushima S (2022). Thread-like appendix on *Arisaema urashima* (Araceae) attracts fungus gnat pollinators. Ecology.

[CR45] Suetsugu K, Okada H, Hirota SK, Suyama Y (2022). Evolutionary history of mycorrhizal associations between Japanese *Oxygyne* (Thismiaceae) species and Glomeraceae fungi. New Phytol.

[CR46] Suetsugu K, Yamana K, Okada H (2023). Rediscovery of the presumably extinct fairy lantern *Thismia kobensis* (Thismiaceae) in Hyogo Prefecture, Japan, with discussions on its taxonomy, evolutionary history, and conservation. Phytotaxa.

[CR47] Tabei Y, Kiryu H, Kin T, Asai K (2008). A fast structural multiple alignment method for long RNA sequences. BMC Bioinformatics.

[CR48] The Angiosperm Phylogeny Group (2016). An update of the Angiosperm Phylogeny Group classification for the orders and families of flowering plants: APG IV. Bot J Linn Soc.

[CR49] Thiers B (2023) Index Herbariorum: A Global Directory of Public Herbaria and Associated Staff. New York Botanical Garden’s Virtual Herbarium. http://sweetgum.nybg.org/science/ih/. Accessed 6 Dec 2023

[CR50] Thorogood CJ (2019). *Oxygyne*: An extraordinarily elusive flower. Plants People Planet.

[CR51] Thorogood CJ, Mat Yunoh S-M (2021). Fairy lanterns in focus. Plants People Planet.

[CR52] White TJ, Bruns T, Lee S, Taylor J, Innis MA, Gelfand DH, Sninsky JJ, White TJ (1990). Amplification and direct sequencing of fungal ribosomal RNA genes for phylogenetics. PCR protocols: a guide to methods and applications.

[CR53] Woodward C, Berry PE, Maas-van de Kamer H, Swing K (2007). *Tiputinia foetida*, a new mycoheterotrophic genus of Thismiaceae from Amazonian Ecuador, and a likely case of deceit pollination. Taxon.

[CR54] Yang S-Z, Lin J-S, Hsu C-J (2010). Morphological characteristics of flower and seed coat of the endangered species of *Thismia taiwanensis* (Burmanniaceae). Taiwania.

[CR55] Yokoyama J, Koizumi Y, Yokota M, Tsukaya H (2008). Phylogenetic position of *Oxygyne shinzatoi* (Burmanniaceae) inferred from 18S rDNA sequences. J Plant Res.

