# Supplementary material for: Relictithismia kimotsukiensis, a new genus and species of Thismiaceae from southern Japan with discussions on its phylogenetic relationship
Source: J Plant Res. 2024 Feb 29;137(3):411–22. doi: 10.1007/s10265-024-01532-5 (PMC11082003; doi:10.1007/s10265-024-01532-5)
Supplement: Supplementary file 1 — Supplementary file1 (PDF 133 KB) [file 10265_2024_1532_MOESM1_ESM.pdf]

Electronic Supplementary Material

Relictithismia kimotsukiensis, a new genus and species of Thismiaceae from southern Japan with discussions on its phylogenetic relationship

Kenji Suetsugu, Yasunori Nakamura, Takafumi Nakano, Shuichiro Tagane

Corresponding author:

Kenji Suetsugu
Department of Biology, Graduate School of Science, Kobe University, Kobe 657-8501, Japan
Email: kenji.suetsugu@gmail.com

Shuichiro Tagane
The Kagoshima University Museum, Kagoshima University, 1-21-30, Korimoto, Kagoshima, 890-0065, Japan
E-mail: stagane29@gmail.com

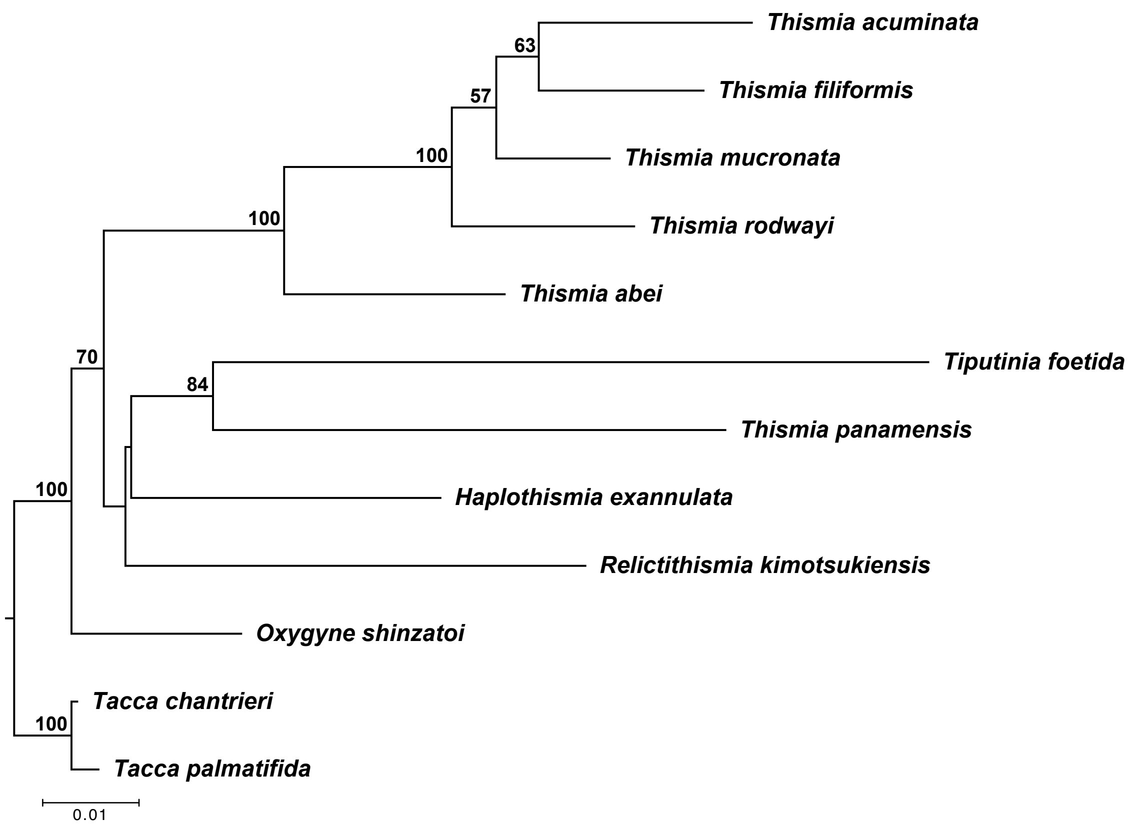

Fig. S1 Maximum-likelihood phylogenetic tree of the combined 18S rDNA and *atpA* sequences from *Relictithismia kimotsukiensis* and its related taxa. Node values indicate bootstrap support (1000 replicates) with a threshold of ≥ 50%. The scale bar represents the number of substitutions per site.

Table S1. Information on species, voucher specimens, and INSD accession numbers used for phylogenetic analysis.

species	specimen voucher	18S	*atpA*	notes
Relictithismia kimotsukiensis	*Y. Nakamura & S. Tagane 23060901* (TNS: holotype)	LC775771	LC775773	
Relictithismia kimotsukiensis	*Y. Nakamura & S. Tagane 23060901* (KYO: isotype)	LC775772	LC775774	
Thismia abei	*K. Suetsugu s.n.* (KYO)	MK356115	MN072714	Old World *Thismia* Clade: Clade 1 *sensu* Shepeleva et al. (2020)
Thismia rodwayi	*Merckx & Wapstra TAS3-1* (L)	KF692536	KF692540	Old World *Thismia* Clade: Clade 2 *sensu* Shepeleva et al. (2020)
Thismia acuminata	*M. Sochor, M. Hroneš, M. Dančák, Z. Egertová & D. Atu BOR6/17* (SAR)	MG008350	MG008365	Old World *Thismia* Clade: Clade 3 *sensu* Shepeleva et al. (2020)
Thismia mucronata	*M.S. Nuraliev 813* (MW)	MK356106	MN072707	Old World *Thismia* Clade: Clade 4 *sensu* Shepeleva et al. (2020)
Thismia filiformis	S. Chantanaorrapint & C. Promma 3928 (PSU)	MK356103	MN072706	Old World *Thismia* Clade: Clade 5 *sensu* Shepeleva et al. (2020)
Thismia panamensis	*Aizprua 2946* (LV)	DQ786081	EU421050	
Tiputinia foetida	*Alvaro Javier Perez Castaneda s.n.* (LV)	FJ215764	FJ215770	
Haplothismia exannulata	*Sasidharan & Sujanapal 30476* (KRFI)	DQ786082	EU421037	
Oxygyne shinzatoi	*M. Yokota s.n.* (RYU)	AB437090	NA	
Tacca chantrieri	*Chase 175* (NCU)	DQ786086	EU421044	Outgroup of Thismiaceae
Tacca palmatifida	Bogor BG, in cult.	DQ786084	FJ215774	Outgroup of Thismiaceae
